# In‐depth proteomics reveals the characteristic developmental profiles of early lung adenocarcinoma with epidermal growth factor receptor mutation

**DOI:** 10.1002/cam4.5766

**Published:** 2023-04-02

**Authors:** Tomoko Dai, Jun Adachi, Yuichi Dai, Noriyuki Nakano, Mariko Yamato, Shinji Kikuchi, Shingo Usui, Yuko Minami, Takeshi Tomonaga, Masayuki Noguchi, Daisuke Matsubara, Noriaki Sakamoto

**Affiliations:** ^1^ Tsukuba Human Tissue Biobank Center University of Tsukuba Hospital Ibaraki Japan; ^2^ Tsukuba Human Tissue Diagnostic Center University of Tsukuba Hospital Ibaraki Japan; ^3^ Laboratory of Proteome for Drug Discovery Center for Drug Design Research National Institute of Biomedical Innovation, Health and Nutrition Osaka Ibaraki Japan; ^4^ Department of Pathology Tsukuba Memorial Hospital Ibaraki Japan; ^5^ Department of Pathology National Center for Child Health and Development Tokyo Japan; ^6^ Department of Pathology University of Tsukuba Hospital Ibaraki Japan; ^7^ Department of Thoracic Surgery, Faculty of Medicine University of Tsukuba Ibaraki Japan; ^8^ Department of Thoracic Surgery Ibaraki Prefectural Central Hospital Ibaraki Japan; ^9^ Department of Respiratory Surgery and Pathology National Hospital Organization Ibaraki Higashi National Hospital The Center of Chest Diseases and Severe Motor & Intellectual Disabilities Ibaraki Japan; ^10^ Department of Pathology Narita Tomisato Tokushukai Hospital Chiba Japan; ^11^ Center of Clinical and Translational Science Shonan Kamakura General Hospital Kamakura Japan; ^12^ Department of Diagnostic Pathology, Faculty of Medicine University of Tsukuba Ibaraki Japan

**Keywords:** early lung adenocarcinoma, EGFR mutation, invasive lung adenocarcinoma, proteomics, stepwise progression

## Abstract

**Introduction:**

Lung adenocarcinoma progresses stepwise from atypical adenomatous hyperplasia to adenocarcinoma in situ (AIS), followed by minimally invasive adenocarcinoma (MIA), and then obvious invasive adenocarcinoma. In this study, we examined the protein expression profiles of early and epidermal growth factor receptor (EGFR) mutation‐positive lung adenocarcinomas.

**Methods:**

Fifteen cases of small and EGFR mutation‐positive adenocarcinomas were collected, including AIS, MIA, and small invasive adenocarcinoma (SIA). We examined their protein expression profiles by tandem mass tag (TMT)‐labeling liquid chromatography‐mass spectrometry (LC–MS/MS) and compared the results between AIS and MIA versus SIA. The differentially expressed proteins were then verified by Western blot analysis and immunohistochemistry (IHC). The clinicopathological implications of the proteins were also examined by IHC.

**Results:**

A total of 4220 proteins were identified by LC–MS/MS analysis. Pathway analysis of the differentially expressed proteins revealed that pathways related to interferon α/β signaling, glutamate and glutamine metabolism, and gluconeogenesis were upregulated in SIA relative to AIS. Among the 13 differentially expressed proteins, cellular retinoic acid binding protein 2 (CRABP2), delta(24)‐sterol reductase (DHCR24), and adenylate kinase 4 (AK4) were expressed significantly more strongly in SIA than in AIS. Patients with high expression of CRABP2, DHCR24, and AK4 showed a significantly poorer outcome than those with low expression.

**Conclusion:**

In comparison with AIS, SIA shows differences in several different protein expression pathways. Furthermore, CRABP2, DHCR24, and AK4 are useful IHC markers for diagnosis of lung adenocarcinoma invasiveness and may be associated with malignant progression of AIS.

## Graphical abstract

Lung adenocarcinoma progresses stepwise from atypical adenomatous hyperplasia (AAH) to adenocarcinoma in situ (AIS), followed by minimally invasive adenocarcinoma (MIA), and then obvious invasive adenocarcinoma. In this study, we examined the protein expression profiles of early and epidermal growth factor receptor (EGFR) mutation‐positive lung adenocarcinomas. In comparison with AIS, SIA (small invasive adenocarcinoma) shows differences in several different protein expression pathways. Furthermore, CRABP2, DHCR24, and AK4 are useful IHC markers for diagnosis of lung adenocarcinoma invasiveness and may be associated with malignant progression of AIS.

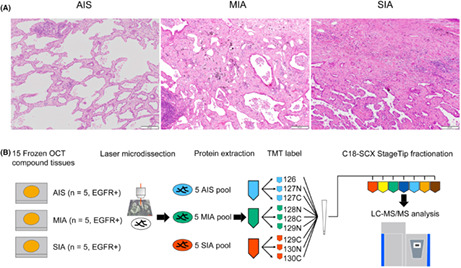

## 1 INTRODUCTION

Lung cancer is the leading cause of cancer death worldwide.^1^ Lung adenocarcinoma is the most common subtype and its incidence is increasing in many countries.^2^ Although recent large‐scale multi‐omics analysis^3^ and multi‐regional sequencing^4^ have led to a broad understanding of the malignant progression of lung adenocarcinoma, details of the molecular mechanism of malignant progression in early‐stage lung adenocarcinoma remain unknown.

In 1995, Noguchi et al. examined small lung adenocarcinomas (<2 cm in diameter) clinicopathologically and divided them into two groups: replacement type and non‐replacement type.^5^ Replacement‐type adenocarcinoma develops from the terminal respiratory unit (TRU) of the peripheral lung parenchyma, and comprises three types: A, B, and C. Noguchi types A and B adenocarcinomas correspond to adenocarcinoma in situ (AIS), whereas Noguchi type C adenocarcinoma includes active fibroblastic proliferation (cancer stroma) and behaves as invasive adenocarcinoma.^5^ On this basis, peripheral‐type lung adenocarcinoma is thought to develop stepwise from atypical adenomatous hyperplasia (AAH), AIS (Noguchi type A, B), and minimally invasive adenocarcinoma (MIA) to early invasive adenocarcinoma (Noguchi type C).^6^, ^7^

On the other hand, many abnormalities of driver oncogenes, such as EGFR, RAS, ALK, MET, ROS, RET, NTRK, and BRAF have been reported,^8^ and many inhibitors against these oncogenic proteins are now being prescribed clinically.^9^ Among various driver oncogenes, epidermal growth factor receptor (EGFR) mutations have been detected even in very early and small lung adenocarcinomas, such as MIA and AIS.^10^, ^11^, ^12^, ^13^ In 2007, Sakamoto et al. reported that the pneumocytes covering the TRU acquire proliferative activity and progress to adenocarcinoma through EGFR mutation.^14^ However, details of the molecular mechanism of malignant progression after acquisition of EGFR mutation have remained unclear.

Using targeted next‐generation sequencing, Qian et al. examined AIS, MIA, and early invasive adenocarcinoma (ADC) and found that although AIS and ADC shared many significant gene mutations, the burden of deleterious mutations was significantly greater in ADC than in AIS.^11^ In addition, multi‐region exome sequencing data for AAH, AIS, MIA, and ADC in Japanese and Chinese cohorts have indicated that EGFR mutation is a minor subclone in AAH, but a major one in AIS, MIA, and ADC.^12^ Furthermore, Kobayashi et al. reported the presence of EGFR mutation in 64% of adenocarcinomas presenting as ground‐glass nodules (GGNs), and noted that EGFR mutation‐positive GGNs showed tumor growth to a greater degree than EGFR mutation‐negative GGNs.^15^ These findings suggested that EGFR mutation is detectable in very early‐stage adenocarcinomas and that EGFR mutation‐positive AIS might progress uniquely and independently in lung adenocarcinomas.

Recently, highly sensitive mass spectrometers^16^ and quantitative labeling reagents^17^ have been developed, facilitating deep quantitative proteomic analysis. In the present study, using the improved Stage Tip fraction method,^18^ we examined and compared the protein expression profiles of AIS, MIA, and small invasive adenocarcinoma (SIA) using small amounts of protein obtained by laser microdissection from small lung adenocarcinomas. We were able to identify characteristic protein pathways that were differentially expressed between AIS and SIA. We also identified several proteins that could be potentially useful markers for diagnosis of malignancies and might affect the malignant progression of EGFR‐positive early lung adenocarcinomas.

## 2 MATERIALS AND METHODS

### 2.1 Tissue samples

All clinical samples were collected retrospectively. For liquid chromatography‐mass spectrometry (LC–MS/MS) analysis, we collected 15 specimens including AIS (*n* = 5), MIA (*n* = 5), and SIA with poor outcome (*n* = 5) from adenocarcinomas that had been surgically resected at the University of Tsukuba Hospital (Ibaraki, Japan) or Ibaraki Higashi National Hospital (Ibaraki, Japan) between 2003 and 2015. Table 1 shows the clinical information for the cases included in this study. The 5‐year survival rate for the AIS and MIA cases was 100% without any metastasis, whereas in the SIA cases, lymph node metastasis or distant metastasis had been identified at the time of surgery or within 5 years after surgery. The SIAs were diagnosed as Noguchi type C adenocarcinoma. All of the cases harbored EGFR mutation (Table 1). Tissues were embedded in optimal cutting temperature (OCT) compound and stored at −80°C. This study was approved by the ethics committees of the University of Tsukuba Hospital (No. H27‐205) and Ibaraki Higashi National Hospital (2015‐018). Written informed consent was obtained from all patients.

**TABLE 1:** Clinical information on patients who underwent LC–MS/MS analysis.

Subtype	AIS (*n* = 5)	MIA (*n* = 5)	SIA (*n* = 5)
Age (years), median	61	65	72
Sex ratio M/F	2/3	2/3	2/3
pstage			
0	5	0	0
I	0	5	2
III	0	0	3
Lymph node metastasis pN ≧1	0	0	3
Metastasis within 5 years	0	0	5
EGFR Ex21 L858R	3	2	3
Ex19 del	2	3	2

Abbreviations: AIS, adenocarcinoma in situ; MIA, minimally invasive adenocarcinoma; SIA, small invasive adenocarcinoma.

For Western blot analysis, we collected eight lung adenocarcinomas that had been surgically resected at the University of Tsukuba Hospital between 2013 and 2017. Among them, five cases were the same as those used for LC–MS/MS analysis.

To confirm the results of LC–MS/MS analysis, we examined 44 lung adenocarcinomas using immunohistochemistry (IHC). The adenocarcinomas had been surgically resected at the University of Tsukuba Hospital or Ibaraki Higashi National Hospital between 2002 and 2017. Tissues were fixed routinely in formalin and embedded in paraffin (FFPE). The 15 cases examined by LC–MS/MS were included among the 44 adenocarcinomas.

To investigate the relationship between the level of protein expression and prognosis, we constructed a tissue microarray (TMA) using 169 lung adenocarcinomas that had been surgically resected at the University of Tsukuba Hospital between 1999 and 2007. The specimens had been histologically diagnosed in accordance with the WHO classification (5th edition) and the UICC TNM classification of malignant tumors (8th edition).

The EGFR mutation analysis was performed using PNA‐LNA PCR clamp method (LSI Medience).

### 2.2 Laser microdissection

Frozen tissue embedded in OCT compound was sliced at a thickness of 10 μm and mounted on PET‐membrane frame slides (Leica Microsystems). The slides were fixed with 70% ethanol and stained with hematoxylin. An area of approximately 10 mm^2^ in each case was microdissected using a LMD6000 (Leica Microsystems). We performed LMD for all tumor cells in both the invasive and lepidic parts of MIA and SIA (Figure S1).

### 2.3 Protein extraction and TMT labeling

Microdissected tumor tissue from five cases of each type (AIS, MIA, and SIA) was mixed and pooled. Details are provided in Supplementary Methods. Protein from each pool of tumor tissue was extracted using MPEX PTS Reagent (GL Science). Twenty micrograms of protein lysate for each subtype was divided equally into three for triplicate experiments and digested with 1:50 (w/w) trypsin (Roche). Peptides of each subtype were labeled with tandem mass tag (TMT) 10 plex reagents (Thermo Fisher Scientific) in accordance with the manufacturer's protocol. Nine samples were combined and dried by SpeedVac (Thermo Fisher Scientific).

### 2.4 Fractionation with C18‐SCX Stage Tip and LC–MS/MS analysis

Each TMT‐labeled sample was fractionated by C18‐SCX Stage Tip into seven parts as described previously.^18^ LC–MS/MS analysis was performed using a Q‐Exactive Plus mass spectrometer (Thermo Fisher Scientific) with an Ultimate 3000 nano HPLC system (Thermo Fisher Scientific) and an HTC‐PAL autosampler (CTC Analytics). The data were analyzed using Proteome Discoverer (Thermo Fisher Scientific). Detailed information on peptide search parameters is provided in Supplementary Methods.

### 2.5 Western blotting

Total protein was extracted from frozen tissues using T‐PER reagent (Thermo Fisher Scientific) containing Halt protease and phosphatase inhibitor cocktail (Thermo Fisher Scientific) and separated on Mini‐PROTEAN TGX gel (Bio‐Rad). The antibodies used are described in Supplementary Methods. Band intensity was quantified using Image Lab software (Bio‐Rad).

### 2.6 Immunohistochemistry

Tissues were sliced at 3 μm thickness from FFPE blocks. Details of the IHC methods are described in Supplementary Methods. The sections were incubated with rabbit monoclonal antibodies against CRABP2 (1:1000), DHCR24 (1:175), and AK4 (1:800), which were the same antibodies as those used for Western blotting.

We evaluated the cytoplasmic staining of CRABP2 and DHCR24, and calculated the results by *H*‐score, as described previously.^19^

### 2.7 Data analysis

HLA histocompatibility antigens were excluded because they vary from individual to individual and the samples were pooled. Median‐centered log 2 values of individual protein abundance were used for principal component analysis (PCA), hierarchical clustering, and parallel coordinate plots.

PCA was performed with the FactoMineR and factoextra packages in R studio software version 1.3.1093 using R^20^ version 4.0.3.

Hierarchical clustering was performed using the pheatmap R package with euclidean distance as the distance and ward.D2 as the linkage function. Three clusters were obtained by cutting the dendrogram using the cutree R function. Proteins differentially expressed among the three subtypes (AIS vs. MIA, MIA vs. SIA, or SIA vs. AIS) were selected using the Kruskal–Wallis test followed by Dunn's test (*p* < 0.05) with SPSS 26 (IBM). Proteins showing 1.5× or greater expression were used to find differences in pathway clustering between AIS, MIA, and SIA. Enrichment analysis of the three clusters was performed by Reactome pathway analysis.^21^

Proteins showing a fold change of ≥1.5 between AIS and SIA were visualized using parallel coordinate plots with GGally in R.

Volcano plots were visualized using GraphPad prism version 9.2.0 (GraphPad Software). In the volcano plot, all proteins were plotted; proteins showing twofold or greater expression were considered to be differentially expressed in AIS and SIA with Welch's *t*‐test.

Statistical analysis of comparisons for Western blot was performed with Mann–Whitney *U* test using GraphPad Prism version 9.2.0.

Statistical analysis of comparisons for the *H*‐score of the three subtypes was performed with Kruskal–Wallis test followed by Dunn's test with GraphPad Prism version 9.2.0.

The receiver operating characteristic (ROC) curve method was used for determining the cutoff point for IHC scoring. Survival curves were calculated using the Kaplan–Meier method and assessed using the log‐rank test by SPSS 26 (IBM). The survival period was from the date of resection to the date of death by any cause. Correlations between clinicopathological features and CRABP2, DHCR24, and AK4 expression were analyzed using the chi‐squared test by SPSS 26 (IBM).

## 3 RESULTS

### 3.1 Quantitative proteomic analysis workflow and sample collection

**FIGURE 1:**
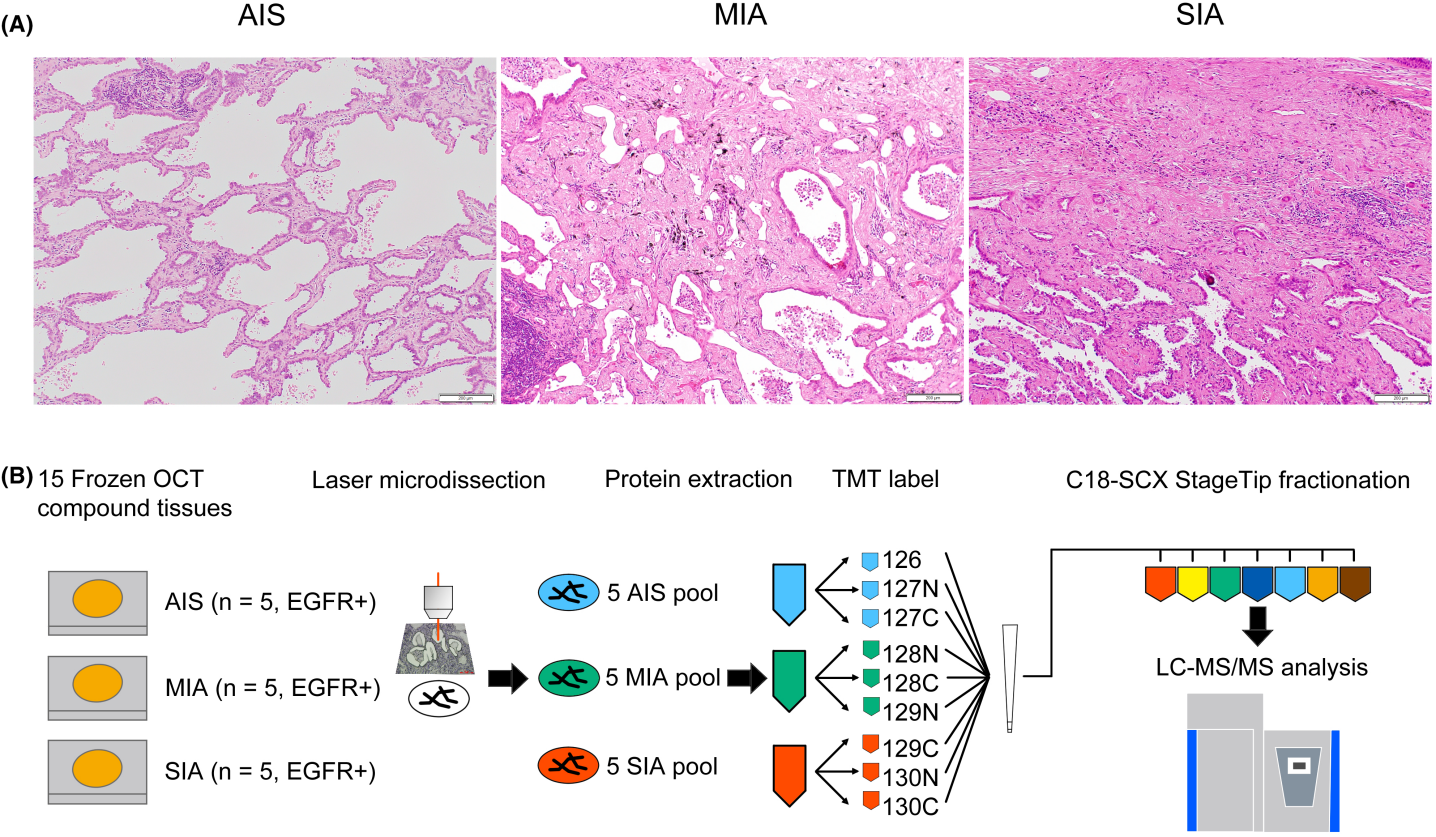
Workflow of quantitative proteomics analysis of small‐sized lung adenocarcinomas harboring EGFR mutation. (A) Representative images of HE staining of adenocarcinoma in situ (AIS), minimally invasive adenocarcinoma (MIA), and small invasive adenocarcinoma (SIA). Five cases of each were examined. (B) We collected tumor cells using laser microdissection from 15 samples of frozen tissues and pooled 5 cases for each subtype. After protein extraction, samples were divided into three parts for triplicate experiments. Digests were TMT labeled and fractioned with C18‐SCX Stage Tip for LC–MS/MS analysis.

In order to compare the protein expression profiles between AIS/MIA and SIA, we conducted quantitative proteomic analysis using EGFR‐mutated AIS (*n* = 5), MIA (*n* = 5), and SIA (*n* = 5) (Figure 1A). Sample information and details of the experimental workflow are given in Table 1 and Figure 1B. All cases subjected to LC–MS/MS analysis contained EGFR mutation (L858R in exon 21 or deletion in exon 19). We selected SIA patients who had developed metastasis within 5 years and/or who had lymph node metastasis at the time of surgery. A total of 4220 proteins were identified (Table S1). After excluding HLA histocompatibility antigens, the number of proteins was finally reduced to 4192.

### 3.2 PCA and enrichment analysis

**FIGURE 2:**
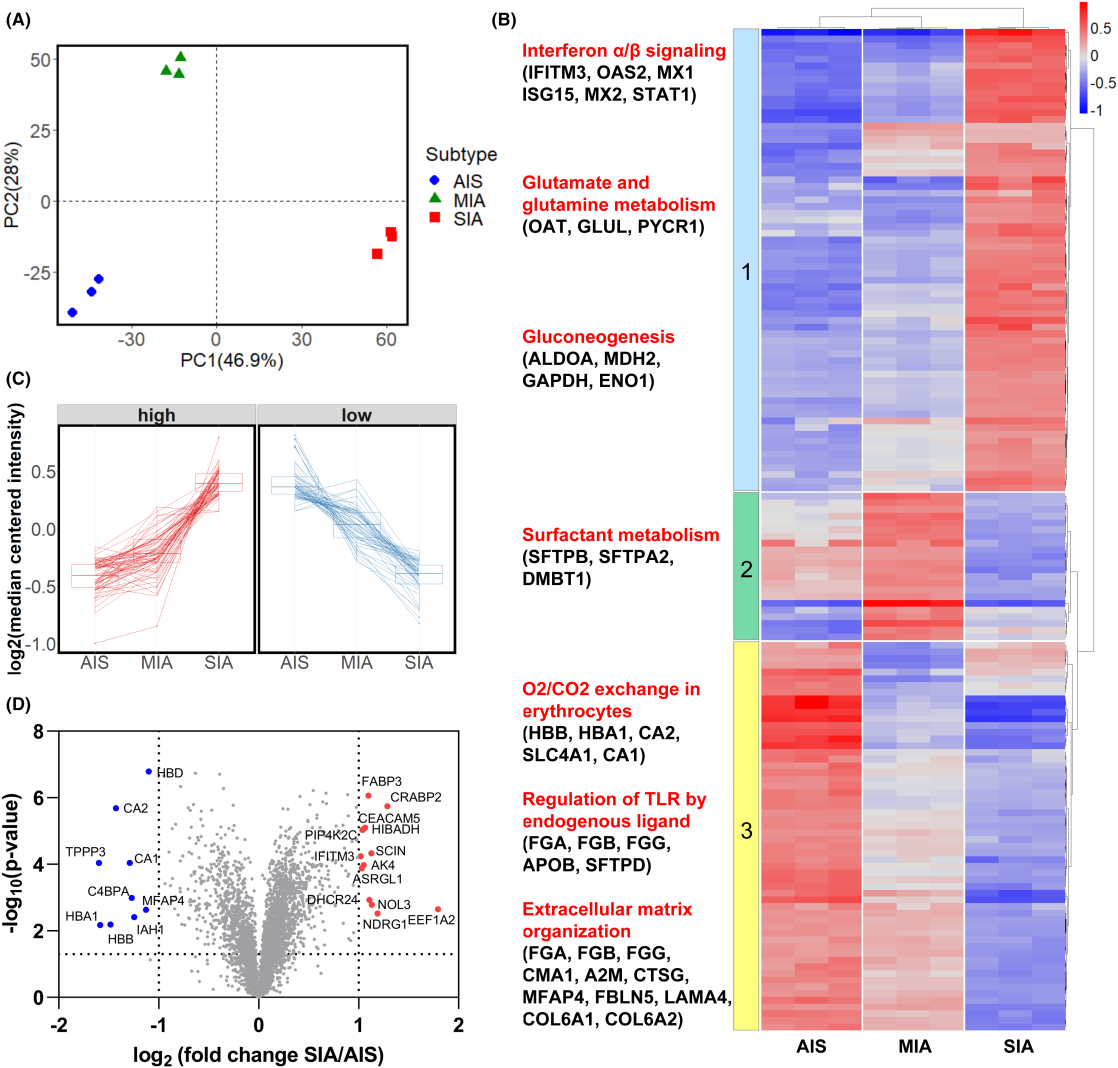
Quantitative proteomics profile of adenocarcinoma in situ (AIS), minimally invasive adenocarcinoma (MIA), and small invasive adenocarcinoma (SIA). (A) Principal component analysis of nine samples was performed using FactoMineR and factoextra in R. (B) Heatmap of proteins (*n* = 149) that differed among AIS, MIA, and SIA. Proteins differentially expressed among the three subtypes were selected using the Kruskal–Wallis test followed by Dunn's test (*p* < 0.05), with a fold change greater than 1.5. Hierarchical clustering was performed using the pheatmap R package. Each protein expression value was the log 2 median centered ratio. Scale bar shows protein expression (red for upregulated, blue for downregulated). Enrichment analysis of three protein clusters was performed by Reactome pathway analysis. (C) Parallel coordinate plot of proteins whose fold change was ≥1.5 between AIS and SIA. Left panel shows proteins that had higher expression in SIA than in AIS. Right panel shows proteins that had lower expression in SIA than in AIS. (D) Volcano plot of all proteins (*n* = 4192) in AIS and SIA. The color dots indicate proteins for which the expression fold change was >2.0 and *p* < 0.05 (Welch's *t*‐test). Proteins that were upregulated in AIS (blue) and SIA (red): AK4, adenylate kinase 4; ASRGL1, isoaspartyl peptidase/l‐asparaginase; C4BPA, C4b‐binding protein alpha chain; CA1, carbonic anhydrase 1; CA2, carbonic anhydrase 2; CEACAM5, carcinoembryonic antigen‐related cell adhesion molecule 5; CRABP2, cellular retinoic acid binding protein 2; DHCR24, delta(24)‐sterol reductase; EEF1A2, elongation factor 1‐alpha 2; FABP3, fatty acid‐binding protein; HBA1, hemoglobin subunit alpha; HBB, hemoglobin subunit beta; HBD, hemoglobin subunit delta; HIBADH, 3‐hydroxyisobutyrate dehydrogenase; IAH1, isoamyl acetate‐hydrolyzing esterase 1; IFITM3, interferon‐induced transmembrane protein 3; MFAP4, microfibril‐associated glycoprotein 4; NDRG1, N‐Myc Downstream Regulated 1. NOL3, nucleolar protein 3; PIP4K2C, phosphatidylinositol 5‐phosphate 4‐kinase type‐2 gamma; SCIN, adseverin; TPPP3, tubulin polymerization‐promoting protein family member 3.

To verify the results of LC–MS/MS analysis, we performed PCA of the expression of the 4192 proteins. Triplicate experiments were performed for each group. As indicated in Figure 2A, AIS and MIA were separated from SIA in the first principal component (PC1).

Next, we performed hierarchical clustering of proteins that were expressed differently among AIS, MIA, and SIA (*n* = 149) and heat mapping of the results. Finally, we identified three clusters of proteins (Figure 2B, Table S2), and reactome pathway analysis was performed for each cluster (Figure 2B; Table S3). The top‐ranked pathways in the first cluster (*n* = 69) were interferon (IFN) α/β signaling, glutamate and glutamine metabolism, and glucogenesis. The expression levels of the proteins included in those pathways increased gradually from AIS to MIA and SIA. On the other hand, the top‐ranked pathways in the third cluster (*n* = 58) included O_2_/CO_2_ exchange in erythrocytes, regulation of Toll‐like receptor (TLR) by endogenous ligand, and extracellular matrix organization. The expression levels of the proteins associated with these pathways decreased gradually from AIS to MIA and SIA.

Figure 2C shows a parallel coordinates plot for proteins whose abundance showed a fold change of ≥1.5 between AIS and SIA. The left panel represents the protein group showing higher expression from AIS to SIA, and the right panel the protein group showing lower expression from AIS to SIA. Most of the proteins showed stepwise upregulation or downregulation from AIS to SIA, or from SIA to AIS.

Volcano plots of AIS and SIA revealed proteins that were more than twice as abundant and significantly different between the two types (Figure 2D). Thirteen proteins (FABP3, CRABP2, CEACAM5, HIBADH, PIP4K2C, IFITM3, SCIN, AK4, ASRGL1, DHCR24, NOL3, NDRG1, and EEF1A2) were significantly upregulated in SIA relative to AIS (Figure 2D).

### 3.3 Western blot analysis of the 13 upregulated proteins

**FIGURE 3:**
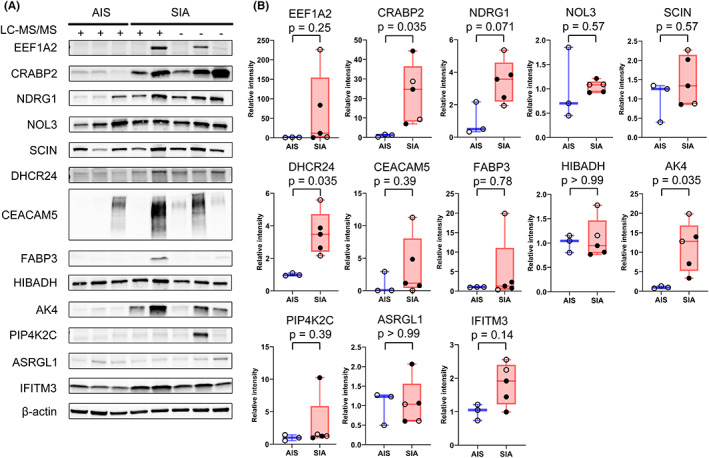
Verification of 13 proteins in adenocarcinoma in situ (AIS) and small invasive adenocarcinoma (SIA) by Western blot analysis. (A) Western blot analysis of AIS (*n* = 3) and SIA (*n* = 5). Three cases of AIS and two cases of SIA were the same as those used for LC–MS/MS analysis. (B) Western blot signal intensity was quantified with Image Lab software. The average SIA intensity was divided by the average for AIS after normalization with β‐actin. Box and whisker plots show median with minimum to maximum range. Blue box shows AIS and red box shows SIA. The same cases as those used for LC–MS/MS analysis are indicated by clear circles. Comparisons were performed using Mann–Whitney *U* test.

Western blotting confirmed that expression of the 13 selected proteins was upregulated (Figure 3). A total of eight frozen specimens of AIS (*n* = 3) and SIA (*n* = 5) containing EGFR mutations (L858R in exon 21 or deletion in exon 19) were examined. Most of them (3 AISs and 2 SIAs) were the same cases as those used for LC–MS/MS analysis, and these are indicated by clear circles in Figure 3B. Among the 13 proteins, CRABP2, DHCR24, and AK4 showed significantly higher expression in SIA than in AIS (*p* = 0.035).

### 3.4 Immunohistochemistry for CRABP2, DHCR24, and AK4

**FIGURE 4:**
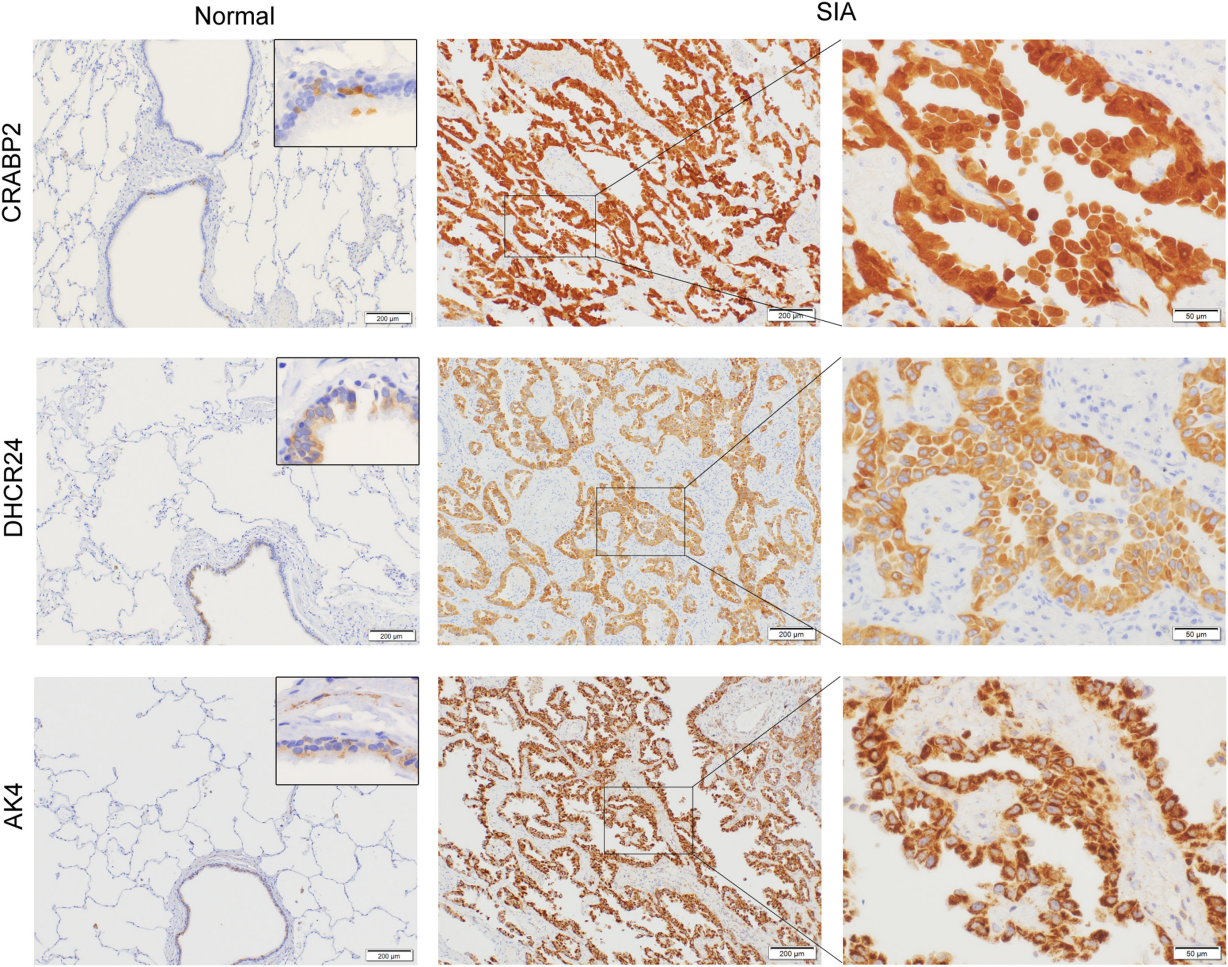
Representative image showing immunohistochemistry for CRABP2, DHCR24, and AK4 in normal lung tissue (left) and SIA (middle and right). Insets are images of normal bronchial epithelium.

**FIGURE 5:**
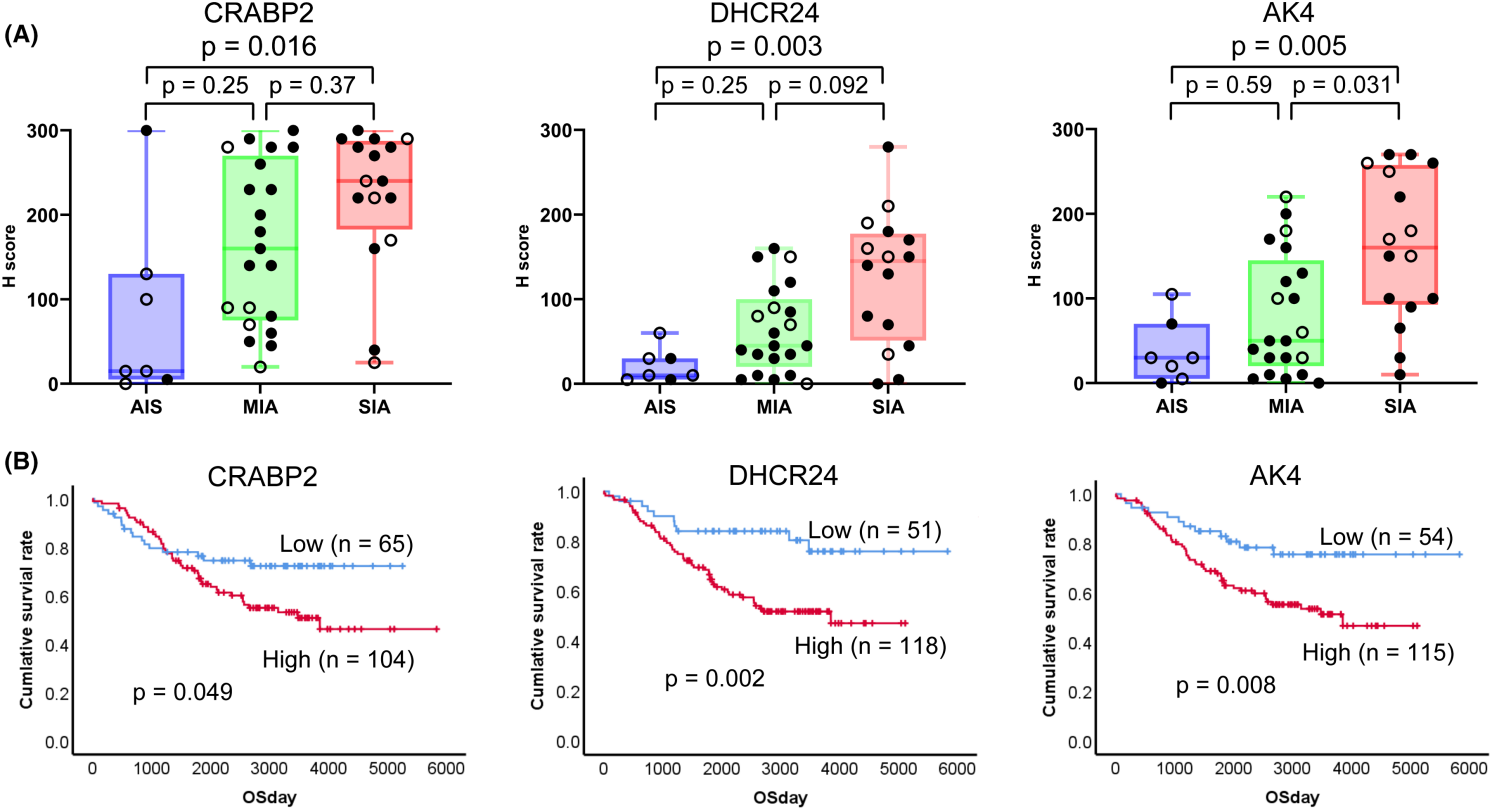
*H*‐scores of immunohistochemistry for CRABP2, DHCR24, and AK4 and their prognostic value. (A) *H*‐scores of CRABP2, DHCR24, and AK4 in adenocarcinoma in situ (AIS) (*n* = 7), minimally invasive adenocarcinoma (MIA) (*n* = 21), and small invasive adenocarcinoma (SIA) (*n* = 16). Box and whisker plots show median with minimum to maximum range. Blue box shows AIS, green box shows MIA, and red box shows SIA. The same cases as those used for LC–MS/MS analysis are indicated by clear circles. Results were compared using Kruskal–Wallis test followed by Dunn's test. (B) Kaplan–Meier analysis of overall survival (log‐rank test in SPSS 26).

Using IHC, the protein expressions of CRABP2, DHCR24, and AK4 were examined using 44 adenocarcinomas including AIS (*n* = 7), MIA (*n* = 21), and SIA (*n* = 16). Figure 4 shows representative histological images of CRABP2, DHCR24, and AK4 in normal lung and SIA. In normal lung tissue adjacent to the tumor, CRABP2, DHCR24, and AK4 were observed in bronchial epithelial cells, but not in alveolar pneumocytes. In tumors, CRABP2 was observed in the nucleus and cytoplasm. DHCR24 was observed in the cytoplasm but not the nucleus. AK4 showed granular staining in the cytoplasm. Consistent with the areas where LMD was performed as described in Section 2, both the invasive and lepidic parts were stained in the same manner. All of CRABP2, DHCR24, and AK4 showed cytoplasmic staining of tumor cells, but no staining was evident in the stroma. The expression intensities of these three proteins were evaluated using the *H*‐score (Figure 5A). There were significant differences between AIS and SIA in the expression of CRABP2 (*p* = 0.016), DHCR24 (*p* = 0.003), and AK4 (*p* = 0.005). AK4 also showed a significant difference (*p* = 0.031) in expression between MIA and SIA. CRABP2, DHCR24, and AK4 showed gradual upregulation from AIS to SIA through MIA (Figure 5A).

### 3.5 Prognostic implications of CRABP2, DHCR24, and AK4 in lung adenocarcinoma

We examined the relationship between the expression levels of CRABP2, DHCR24, and AK4, and patient outcome by IHC using TMA (*n* = 169). By ROC curve analysis, the following cutoff points were determined: CRABP2 (125, sensitivity 0.730 and 0.547 [1 − specificity]), DHCR24 (7.5, sensitivity 0.841 and 0.613 [1 − specificity]), and AK4 (65, sensitivity 0.810 and 0.604 [1 − specificity]). AUC was 0.584 for CRABP2, 0.624 for DHCR24, and 0.612 for AK4, respectively (Figure S2). Cases were divided into high and low expression groups based on the cutoff point. Groups showing high expression of CRABP2 (*p* = 0.049), DHCR24 (*p* = 0.002), and AK4 (*p* = 0.008) had a significantly worse outcome (Figure 5B).

Using chi‐squared test, we assessed the correlation between CRABP2, DHCR24, and AK4 expression and the clinicopathological features of the patients (Table 2). The expressions of CRABP2, DHCR24, and AK4 were significantly correlated with pathological stage, vascular invasion, lymphatic permeation, and histological subtype. Furthermore, the expressions of CRABP2 and AK4 were also significantly correlated with lymph node metastasis.

**TABLE 2:** Expression of CRABP2, DHCR24, and AK4, and clinicopathological features in patients with lung adenocarcinoma.

		CRABP2 expression			DHCR24 expression			AK4 expression			
Clinicopathological features		Low	High	*p‐*Value	Low	High	*p‐*Value	Low	High	*p‐*Value	
Total patients	169	65	104		51	118		54	115		
Age (years)				0.67			0.79			0.58	
≤60	42	15	27		12	30		12	30		
>60	127	50	77		39	88		42	85		
Gender				0.18			0.061			0.076	
Male	101	43	58		25	76		27	74		
Female	68	22	46		26	42		27	41		
Pathological stage (0 vs. others)				0.001			0.001			< 0.001	
Stage 0	19	14	5		12	7		18	1		
Stage I	89	28	61		27	62		25	64		
Stage II	25	9	16		2	23		4	21		
Stage III	33	12	21		9	24		6	27		
Stage IV	3	2	1		1	2		1	2		
Lymph node status				0.018			0.056			0.003	
N0/Nx	126	55	71		43	83		48	78		
N1 and N2	43	10	33		8	35		6	37		
Pleural invasion				0.75			0.065			0.069	
pl0	112	44	68		39	73		41	71		
pl1‐3	57	21	36		12	45		13	44		
Vascular invasion				0.019			0.012			0.01	
V0	98	45	53		37	61		39	59		
V1	71	20	51		14	57		15	56		
Lymphatic permeation				0.042			0.002			0.036	
Ly0	106	47	59		41	65		40	66		
Ly1	63	18	45		10	53		14	49		
Histological subtype (AIS, MIA vs. others)				0.001			0.007			< 0.001	
Adenocarcinoma in situ	19	14	5		12	7		18	1		
Minimally invasive adenocarcinoma	8	4	4		2	6		6	2		
Lepidic	45	7	38		10	35		12	33		
Acinar	18	2	16		3	15		2	16		
Papillary	26	8	18		5	21		9	17		
Micropapillary	3	1	2		0	3		1	2		
Solid	30	14	16		7	23		6	24		
Invasive mucinous	20	15	5		12	8		0	20		
EGFR mutation ( − vs. +)				0.038			0.094			0.013	
−	23	11	12		9	14		13	10		
+	10	1	9		1	9		1	9		

## 4 DISCUSSION

In the present LC–MS/MS analysis, we were able to identify more than 4000 proteins expressed in early‐stage lung adenocarcinoma. Hierarchical clustering and pathway analysis of the identified proteins showed that IFN‐α/β signaling, glutamate and glutamine metabolism, and gluconeogenesis were activated in SIA (Figure 2B). IFN signaling is known to be an anti‐tumor immune response.^22^ scRNA‐seq analysis of lung adenocarcinoma has indicated that the IFN‐α response is enriched in endothelial cells and fibroblasts in tumors, suggesting an association with inflammation.^23^ Endothelial cells and fibroblasts may be included in samples obtained by LMD. IFN‐α/β signaling upregulation might be the result of a cellular anti‐tumor or inflammation response. Several studies of lung adenocarcinoma have suggested glycogenesis in lung cancer cells and the use of glutamine as an energy source.^24^, ^25^ These findings indicate that metabolic reprogramming of cancer cells might be more marked in SIA than in AIS and MIA.

On the other hand, we found that expression of erythrocyte‐related proteins, endogenous ligands of TLR, and extracellular matrix organization were decreased in SIA (Figure 2B). Such a decrease in erythrocyte‐associated proteins might be due to hypoxia caused by compression of intratumoral vessels.^26^ DAMPs (damage‐associated molecular patterns), acting as endogenous ligands for TLRs, have been reported to have both anti‐tumor and tumor‐promoting effects.^27^ Although we were unable to clarify the direction of these effects, our results may indicate that innate immunity is associated with tumor progression from AIS to SIA. Degradation of the extracellular matrix is reported to occur during tumor growth, invasion, and migration.^28^ Extracellular matrix remodeling might occur during progression from AIS to SIA.

As indicated in Figures 3 and 5, we identified three proteins (CRABP2, DHCR24, and AK4) that were more highly expressed in SIA than in AIS. The present clinicopathological studies demonstrated significant differences in the expression of CRABP2, DHCR24, and AK4 in relation to vascular invasion and lymphatic permeation (Table 2), and also significant differences among histological subtypes. High expression of these proteins was significantly associated with unfavorable patient outcome (Figure 5B).

CRABP2 is a retinoic acid (RA) binding protein involved in the transport of RA from the cytoplasm to the nucleus.^29^ It has been reported that CRABP2 is expressed in lung cancer,^30^, ^31^ breast cancer,^32^ and glioblastoma.^33^ In addition, CRABP2 expression is associated with poor survival and recurrence in lung cancer patients.^31^ CRABP2 is localized in glioblastoma, and sequestration of RA in the cytoplasm has been reported to enhance cell proliferation.^33^ In the present study, we evaluated the cytoplasmic staining of CRABP2, but localization was also observed in the nucleus. RA is reported to have both proliferative and growth‐inhibitory functions and is regulated by two related proteins, FABP5 and CRABP2.^34^ Depending on the FABP5/CRABP2 ratio, the effect of RA changes; when CRABP2 is high (low ratio), it tends to inhibit proliferation.^34^ In the present proteomic analysis, FABP5 was included in Cluster 2 of the heatmap and downregulated in SIA (Table S2). These results may indicate that cell proliferation is relatively inhibited in SIA. Further analysis will be needed to determine whether CRABP2 is involved in RA function or has other roles in lung adenocarcinoma.

DHCR24 is an enzyme involved in the biosynthesis of cholesterol,^35^ and its oncogenic activity has been reported in hepatocellular carcinoma^36^ and endometrial carcinoma.^37^ The present study is the first to have investigated the relationship between outcome and protein expression of DHCR24 in lung adenocarcinoma (Figure 5B). Downregulation of DHCR24 has been reported to inhibit lipid raft and caveolar formation^36^, ^38^ through suppression of cholesterol biosynthesis, and thereby tumor growth and invasion.^35^, ^36^, ^37^ Interestingly, a high degree of lipid raft formation has been reported to be associated with resistance to EGFR tyrosine kinase inhibitors (TKIs).^39^ On the other hand, although the role of DHCR24 overexpression in tumor cells is still not clear, it has been reported that DHCR24 protects cells from endoplasmic reticulum stress‐derived apoptosis in neurons, suggesting that it has an anti‐apoptotic function.^40^ Further studies are needed to determine whether a similar anti‐apoptotic mechanism is involved in lung cancer.

AK4 is localized in mitochondria and involved in maintaining the composition of cellular nucleotides by catalyzing the reversible transfer of nucleoside phosphates.^41^, ^42^ AK4 is reportedly expressed in lung adenocarcinoma^43^ and glioma.^44^ As Jan et al. have noted that AK4 downregulates ATF3 and is associated with poor clinical outcome and metastasis,^43^ AK4 might be involved in the malignant progression of lung adenocarcinoma.

We acknowledge that this study had several limitations. First, the samples analyzed using Western blotting included not only the tumor cells but also non‐tumor cells including stroma components (lymphocytes, fibroblasts, vessels, etc.). Especially, although most AISs are composed of tumor cells unassociated with inflammatory cells and fibroblasts, SIAs usually contain infiltrating inflammatory cells and other stromal cells. Second, as the number of AISs, MIAs, and SIAs examined in this study was limited, we cannot rule out the possibility that other important proteins involved in malignant transformation of lung adenocarcinoma might have been present. Third, as we focused on and examined EGFR mutation‐positive adenocarcinomas, it will also be necessary to study EGFR mutation‐negative specimens before any conclusion can be generalized to all lung adenocarcinomas.

In this study of EGFR mutation‐positive early lung adenocarcinoma, the presence of several signaling pathway clusters showing characteristic differences among AISs, MIAs, and SIAs was suggested. Three of the proteins involved (CRABP2, DHCR24, and AK4) were extracted and shown to be associated with patient outcome. The expressions of three proteins can be easily evaluated by IHC and might be potentially useful as markers for indicating the invasiveness of lung adenocarcinoma. These proteins may play important roles in the malignant progression of EGFR mutation‐positive lung adenocarcinoma.

## AUTHOR CONTRIBUTIONS

**Tomoko Dai:** Conceptualization (equal); data curation (lead); formal analysis (lead); funding acquisition (lead); investigation (lead); project administration (equal); visualization (lead); writing – original draft (lead). **Jun Adachi:** Formal analysis (supporting); investigation (supporting); resources (supporting); writing – review and editing (supporting). **Yuichi Dai:** Investigation (supporting); writing – review and editing (supporting). **Noriyuki Nakano:** Investigation (supporting); resources (supporting). **Mariko Yamato:** Investigation (supporting); resources (supporting). **Shinji Kikuchi:** Investigation (supporting); resources (supporting). **Shingo Usui:** Investigation (supporting); resources (supporting). **Yuko Minami:** Investigation (supporting); resources (supporting). **Takeshi Tomonaga:** Resources (supporting). **Masayuki Noguchi:** Conceptualization (equal); project administration (equal); resources (lead); supervision (lead); writing – review and editing (lead). **Daisuke Matsubara:** Resources (supporting). **Noriaki Sakamoto:** Resources (supporting).

## CONFLICT OF INTEREST STATEMENT

The authors have no conflicts of interest to declare.

## ETHICS STATEMENT

This study was approved by the ethics committees of both the University of Tsukuba Hospital (No. H27‐205) and Ibaraki Higashi National Hospital (2015‐018), and conforms to the provisions of the Declaration of Helsinki.

## Supporting information


Figure S1.

Figure S2.

Data S1.

Table S1.

Table S2.

Table S3.

Table S4.

## Data Availability

The data that support the findings of this study are available from the corresponding author upon reasonable request.
