# Supplementary figures and images for: In‐depth proteomics reveals the characteristic developmental profiles of early lung adenocarcinoma with epidermal growth factor receptor mutation
Source: Cancer Med. 2023 Apr 2;12(9):10755–67. doi: 10.1002/cam4.5766 (PMC10225231; doi:10.1002/cam4.5766)

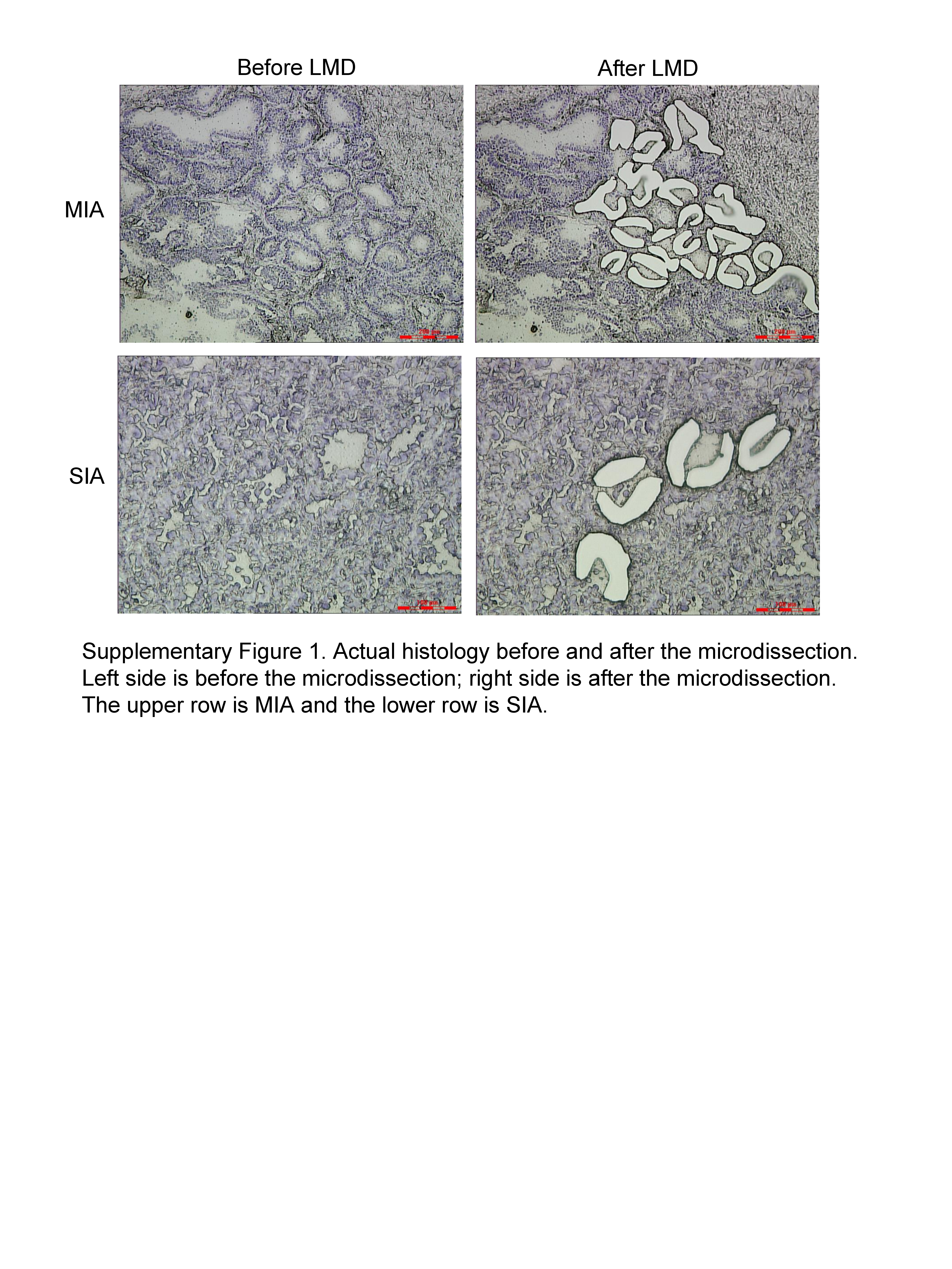

Supplement: Supplementary file 1 — Figure S1. [file CAM4-12-10755-s004.jpg]

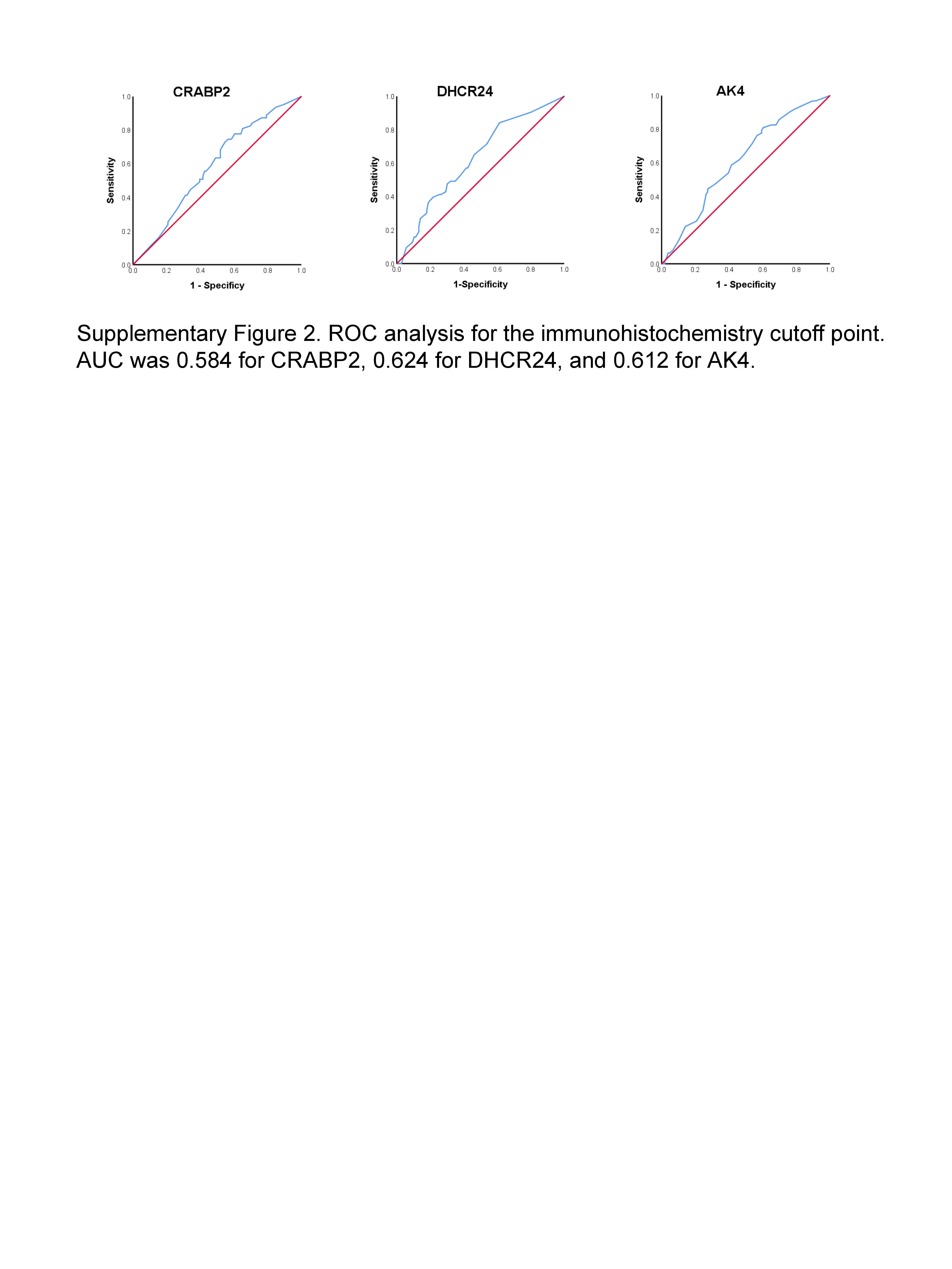

Supplement: Supplementary file 2 — Figure S2. [file CAM4-12-10755-s006.tif]
