# Supplementary material for: In‐depth proteomics reveals the characteristic developmental profiles of early lung adenocarcinoma with epidermal growth factor receptor mutation
Source: Cancer Med. 2023 Apr 2;12(9):10755–67. doi: 10.1002/cam4.5766 (PMC10225231; doi:10.1002/cam4.5766)
Supplement: Supplementary file 3 — Data S1. [file CAM4-12-10755-s001.docx]

**Supplementary Methods**

**Protein extraction and TMT labeling**

Proteins were extracted from tumor cells using MPEX PTS Reagent (GL Science, Tokyo, Japan) containing Complete protease inhibitor cocktail (Roche, Basel, Switzerland). Samples were incubated at 95℃ for 5 min and sonicated for 10 min. The total amount of proteins was measured using Micro BCA protein assay reagent (Thermo Fisher Scientific, Waltham, MA). Protein lysate (20μg) of each subtype was divided equally into three parts for triplicate experiments and digested with 1:50 (w/w) trypsin (Roche, Basel, Switzerland) at 37℃ overnight (Figure 1B). After digestion, samples were acidified with 0.5% trifluoroacetic acid (TFA) and vortexed. After centrifugation at 15,600 x *g* for 3 min, the organic phase was removed and the aqueous phase containing the peptides was collected. Peptides were dried using a SpeedVac (Thermo Fisher Scientific) and redissolved in 2 M urea and 1% TFA. Peptides were desalted on an C18-SCX Stage Tips.

**LC-MS/MS data analysis**

The MS/MS spectra were searched against the UniProt human database with the following search parameters: full tryptic specificity, up to two missed cleavage sites, carbamidomethylation of cysteine residues set as a fixed modification, and N-terminal protein acetylation and methionine oxidation as variable modifications. Search results were filtered to a maximum false discovery rate (FDR) of 0.01 at the protein and peptide levels.

**Antibodies used for Western blotting**

The antibodies used were rabbit polyclonal EEF1A2 antibody (GeneTex, Irvine, CA), rabbit monoclonal CRABP2 antibody (EPR17376, Abcam, Cambridge, UK), rabbit monoclonal NDRG1 antibody (D8G9, Cell Signaling Technology, Danvers, MA), rabbit monoclonal ARC (NOL3) antibody (D7Q3G, Cell Signaling Technology), rabbit monoclonal SCIN antibody (EPR16309, Abcam), rabbit monoclonal DHCR24 antibody (C59D8, Cell Signaling Technology) and mouse monoclonal CEA antibody (CB30, Cell Signaling Technology) for CEACAM5, rabbit monoclonal HIBADH antibody (EPR12519, Abcam) and rabbit monoclonal AK3L1 antibody (EPR7678, Abcam) for AK4, rabbit polyclonal PIP4K2C antibody (Proteintech, Rosemont, IL), rabbit polyclonal ASRGL1 antibody (Cell Signaling Technology), rabbit monoclonal IFITM3 antibody (D8E8G, Cell Signaling Technology) and mouse monoclonal β-actin antibody (Sigma-Aldrich, St. Louis, MO).

**Immunohistochemistry**

Tissues were sliced at 3μm thickness from FFPE blocks. The sections were then deparaffinized. Antigen retrieval was performed using Dako target retrieval solution, pH 9 (Agilent Technologies, Santa Clara, CA) and Dako PTlink at 97°C for 20 min (Agilent Technologies). Immunohistochemistry (IHC) was performed using a Histostainer 36A (Nichirei Biosciences, Tokyo, Japan). The sections were subsequently incubated with the secondary antibody (Dako REAL Envision Detection System, Agilent Technologies) and detected with DAB (Dako DAB+Liquid, Agilent Technologies). The positive control for CRABP2 was normal skin, that for DHCR24 was normal testis, and that for AK4 was normal liver. We evaluated CRABP2, DHCR24, AK4 in the cytoplasm by calculating the H-score, which was defined as the summed percentage of cells at each intensity level of staining: 0 (negative), 1+ (weak), 2+ (intermediate), and 3+ (strong) and calculated using the formula: [1 x (% area 1+) + 2 x (% area 2+) + 3 x (% area 3+)]
