# Supplementary material for: In‐depth proteomics reveals the characteristic developmental profiles of early lung adenocarcinoma with epidermal growth factor receptor mutation
Source: Cancer Med. 2023 Apr 2;12(9):10755–67. doi: 10.1002/cam4.5766 (PMC10225231; doi:10.1002/cam4.5766)
Supplement: Supplementary file 7 — Table S4. [file CAM4-12-10755-s005.docx]

Supplementary Table 4 EGFR information used for WB and IHC

| analysis | WB | | IHC | | |
| --- | --- | --- | --- | --- | --- |
| subtype | AIS (n=3) | SIA  (n=5) | AIS (n=7) | MIA (n=21) | SIA (n=16) |
| EGFR wild type | 0 | 0 | 2 | 7 | 5 |
| EGFR Ex21 L858R | 2 | 5 | 3 | 6 | 8 |
| Ex19 del | 1 | 0 | 2 | 5 | 3 |
| Others  (Ex18 G719A, Ex21 L861Q) | 0 | 0 | 0 | 3 | 0 |
